# Supplementary material for: Effect of Acute Thermal Stress Exposure on Ecophysiological Traits of the Mediterranean Sponge Chondrilla nucula: Implications for Climate Change
Source: Biology (Basel). 2023 Dec 22;13(1):9. doi: 10.3390/biology13010009 (PMC10813260; doi:10.3390/biology13010009)

Supplementary Material

Figure S1. Algae concentration in the clearance rate experiments at the beginning (c1) and the last (c2) experimental time.

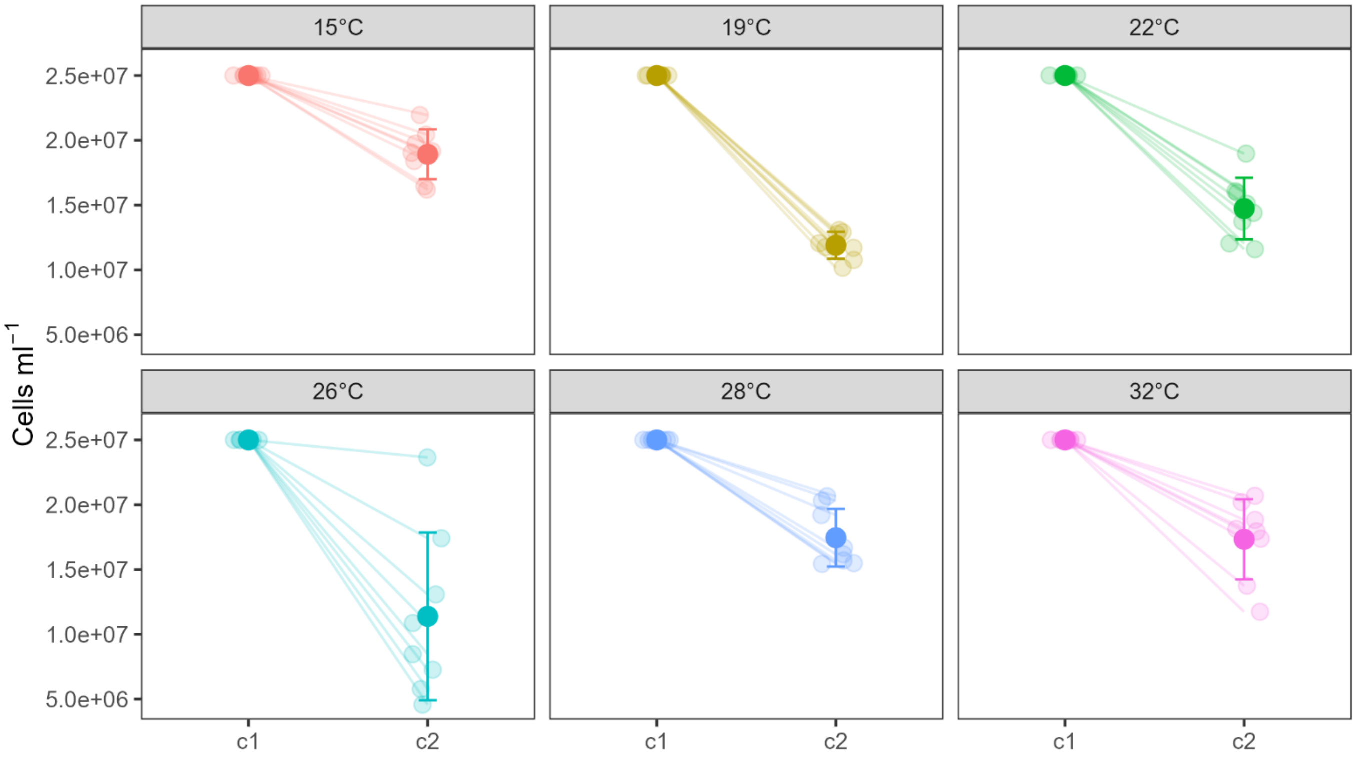

Supplement: Supplementary file 1 [file biology-13-00009-s001.zip › biology-2760169-supplementary.pdf]
